# Temporal Trends in SARS-CoV-2 Antibody Levels Among COVID-19 Patients in Kerala During the First Wave and Pre-vaccination Period

**DOI:** 10.7759/cureus.61650

**Published:** 2024-06-04

**Authors:** Jithu K Mathew, Chandni Radhakrishnan, Ajitha B K, Beena J Philomina, Thulaseedharan N K, Dhananjayan Dhanasooraj

**Affiliations:** 1 Microbiology, Government Medical College, Kottayam, Kottayam, IND; 2 Internal Medicine, Government Medical College, Kozhikode, Kozhikode, IND; 3 Medical Education, Kerala University of Health Sciences, Thrissur, IND; 4 Statistics, Government Medical College, Thrissur, Thrissur, IND; 5 Microbiology, KMCT Medical College, Kozhikode, Kozhikode, IND; 6 Internal Medicine, KMCT Medical College, Kozhikode, Kozhikode, IND; 7 Molecular Biology, Multidisciplinary Research Unit, Government Medical College, Kozhikode, Kozhikode, IND

**Keywords:** sars-cov-2, comorbidities, covid-19 vaccination, sars-cov-2 spike protein, pre-vaccination, first wave, kerala, igg, covid-19, antibody response

## Abstract

Background: The SARS-CoV-2 virus interacts with host cells through the S1 domain of its spike protein. This study measures the IgG immune response to this domain in COVID-19 patients from Kerala, India, and explores its association with various health factors.

Methods: A cohort of 258 COVID-19 patients was analyzed for IgG antibodies targeting the S1 spike protein domain. The temporal pattern of the IgG response and its correlation with hospitalization needs, intensive care, and pre-existing conditions such as diabetes, hypertension, and coronary artery disease were assessed.

Results: A significant IgG response (76.4%) was detected, indicating robust immune activation post-infection. The IgG levels peaked between two to four and four to eight weeks post-infection, with a notable increase at 12 weeks, hinting at possible secondary exposure or an immune memory response. No correlation was found between IgG levels and the presence of diabetes mellitus, hypertension, or coronary artery disease. However, higher IgG responses correlated with the severity of the infection, as seen in patients requiring hospitalization or intensive care.

Conclusions: The IgG response to the S1 spike protein domain serves as a potential marker of immune activation in COVID-19. It reflects the body’s defense mechanism against the virus and may predict disease severity and outcomes. The findings suggest that IgG levels could be indicative of the viral load, inflammatory response, and possibly the likelihood of protection against reinfection.

## Introduction

The coronavirus COVID-19 pandemic is a global crisis that evolved in 2020. The disease was first reported in Wuhan city of China in December 2019, and it rapidly spread to other parts of the world, including Antarctica, and posed a heavy impact on all aspects of the human race [1]. SARS-CoV-2 falls under the genus Betacoronavirus (β-CoV) (family Coronaviridae) virus with a positive-sense ribonucleic acid (RNA) that is packaged in an envelope. The envelope facilitates the virus entry and evasion of the host cell’s defenses. Non-structural proteins of coronaviruses have been shown to impair the function of host innate immune cells and alter cytokine production. Humoral response against SARS-CoV-2 has been found to be similar to that against other coronavirus infections, involving the characteristic IgG and IgM production [2]. B cells respond to the N protein of SARS-CoV early in the infection, whereas S protein-specific antibodies appear after four to eight days of symptom onset. IgA, IgG, and IgM antibodies against SARS-CoV are detected at various time points after symptom onset in infected patients [3]. IgM and IgA antibodies were detected five days after the onset of initial symptoms, whereas IgG was detected after 14 days [4].

In Kerala, the first COVID-19 case emerged in January 2020. Subsequently, the state experienced several waves of infection, each characterized by distinct variants. In this study, we analyzed the IgG response to the S1 domain of the spike protein of SARS-CoV-2, spanning from October 2020 to September 2021, utilizing a commercially available enzyme-linked immunosorbent assay (ELISA) in subjects diagnosed with COVID-19. The study was specifically focused on individuals who had developed natural naive immunity to COVID-19, excluding those who had been vaccinated and those cases of reinfection. Apart from a few selected centers, comprehensive data on the antibody response against COVID-19 from Kerala during the pandemic period are sparse [5].

## Materials and methods

This is a hospital-based cross-sectional study from October 2020 to September 2021 assessed in the Departments of Medicine and Microbiology, Government Medical College, Kozhikode, to measure the IgG antibody response in patients diagnosed with SARS-CoV-2 infection. The objective of this study was to investigate the dynamics and determinants of the antibody response to SARS-CoV-2 infection in a cohort of patients with or without different comorbidities. Our centre was providing diagnostic and treatment services during the pandemic. The subjects were identified from the institutional records and approached for participation in the study. Patients with a confirmed diagnosis of SARS-CoV-2 infection between two weeks and 17 weeks from the day of onset of symptoms or the diagnosis (in asymptomatic cases) were identified. Patients who had received convalescent plasma therapy or received vaccination were excluded.

After obtaining the written informed consent, a structured questionnaire was used to collect demographic and clinical details from 258 laboratory-confirmed (by rapid antigen test, reverse transcription-polymerase chain reaction (RT-PCR), TrueNat, or cartridge-based nucleic acid amplification test (CBNAAT)) COVID-19 subjects.

The patient data were meticulously documented, including comorbidities, exhibited symptoms, the duration of the illness, and patient outcomes. The Clinical Severity Stage was categorized into four distinct levels: asymptomatic, mild, moderate, and severe. The ‘Mild’ category is defined by a respiratory rate lower than 24 breaths per minute and an oxygen saturation (SpO_2_) greater than 94% in ambient air. The ‘Moderate’ category is specified by a respiratory rate between 24 and 29 breaths per minute and SpO_2_ ranging from 91% to 94% in ambient air. The ‘Severe’ category is marked by a respiratory rate of 30 breaths per minute or higher and SpO_2_ below 90% in ambient air. These classifications provide a structured approach to assessing the severity of clinical presentations [6].

From each study participant, 2 mL of plain blood was withdrawn. The sera were then isolated, adhering to established protocols. Upon separation, these sera samples were preserved in a deep freezer (-80°C), maintaining their condition until the ELISA testing phase commenced. ELISA was performed adhering to the kit manufacturer’s instructions. The IgG antibodies against SARS-CoV-2 in serum were measured by a commercially available ELISA kit (EUROIMMUN anti-SARS-CoV-2 ELISA). In brief, the ELISA reagent wells were coated with the S1 domain of the SARS-CoV-2 spike protein, expressed recombinantly in HEK 293 cells. Diluted samples (1:101) and controls were added to microplate wells and incubated. After washing thrice with the provided wash buffer, peroxidase-labeled anti-human IgG was added and incubated. Chromogen/substrate solution was added then and incubated at room temperature as per the suggested protocol. Colour intensity was measured in an ELISA reader (ALTA ELISA Plate Reader; Swastika Ventures, West Bengal, India) at 450 nm, followed by adding a Stop solution. The results were analyzed and interpreted in accordance with the recommendations provided by the kit manufacturers.

The optical density (OD) of each sample was divided by the OD of the calibrator to obtain a ratio. This ratio was then used to categorize the results: values below 0.8 indicated a negative result, values between 0.8 and 1.1 were considered borderline, and values of 1.1 or above signified a positive result. Statistical analysis was performed using Statistical Product and Service Solutions (SPSS, version 16.0; IBM SPSS Statistics for Windows, Chicago, IL). Continuous data were reported as mean ± SD for symmetric data and as median with interquartile range (IQR) for skewed data. Categorical data were reported as frequency and percentages. Since for both positive and negative response groups the data on time after the onset of infection did not meet the normality assumption, the average time after the onset of infection between positive and negative response groups was compared by the Mann-Whitney U test. To test the association between antibody response and co-morbidities and other known factors, the chi-square test was used when all cells had expected frequency > 5. For data not satisfying this condition, Fisher’s exact test was used for testing the association. The correlation between time from the onset of infection and antibody response as titre was assessed by Pearson’s correlation. Two-sided tests were used, and the significance level was set at p<0.05.

## Results

There were 258 patients in our study sample. The time after the onset of infection was between 15 and 119 days with a mean time of 53.8 ± 27.1 days. The median age of the patients was 32 (25-45) years (between two and 93 years). The antibody response (expressed as a ratio) was between 0.003 and 9.07, with a mean of 2.94 ± 2.13. Antibody response was positive in 197 (76.4%) and negative in 44 (17.1%). A total of 13 (5%) had a borderline response, while in four (1.6%) samples, the antibody response was indeterminate. Borderline and indeterminable responses were excluded from the analysis. A total of 68 patients received treatment in the intensive care unit (ICU). Oxygen therapy was administered to 160 individuals. The rate of IgG-positive response showed an increasing trend with the severity of the disease (Table 1). Furthermore, the study found no significant association between comorbid conditions and antibody response (Table 2).

**Table 1:** Relation between antibody response and severity of illness n= number of subjects. Positive, Negative: Samples were compared to the calibrator using optical density ratios; below 0.8 was negative, 0.8-1.1 borderline, and above 1.1 positive.

Sl No.	Severity of illness (n)	Antibody response in number of subjects, n (%)	
		Positive	Negative
1	Asymptomatic (n=45)	28 (62.2)	17 (37.8)
2	Mild (n=140)	116 (82.9)	24 (17.1)
3	Moderate (n=29)	26 (89.7)	3 (10.3)
4	Severe (n=27)	27 (100)	0

**Table 2:** Relation between antibody response and the presence of comorbidities DM: diabetes mellitus, HTN: hypertension, CAD: coronary artery disease. *Significant at 5% level

Presence of comorbidities		Antibody response in number of subjects, n (%)		p value*
		Positive	Negative	
DM	Yes	26 (86.7)	4 (13.3)	0.456
	No	171 (81.0)	40 (19.0)	
HTN	Yes	23 (88.5)	3 (11.5)	0.432
	No	174 (80.9)	41 (19.1)	
CAD	Yes	11 (91.7)	1 (8.3)	0.7
	No	186 (81.2)	43 (18.8)	

**Figure 1:**
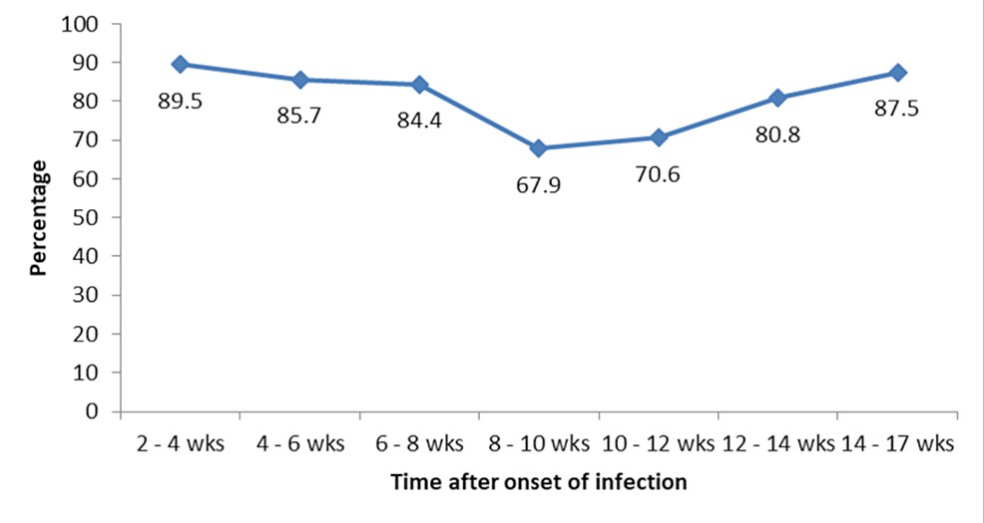
Trend in the positive response rates with reference to time after the onset of infection wks: weeks. Percentage: positive antibody response in subjects

The positive response was significantly higher among patients who required oxygen therapy (Table 3). There was a significant negative correlation between time after the onset of infection and antibody response (r=-0.278, p<0.001). The median time from the onset of infection to antibody estimation was longer in patients with a negative response than with a positive response though could not attain statistical significance (57.5 (42-78) days vs 47 (26-73) days, p=0.061) (Table 4). At 8-12 weeks, the positive response rate was significantly less compared to the positive response rate at two to four weeks, but the same increased from 12 weeks (Figure 1).

**Table 3:** Relation between antibody response in the number of subjects and other known factors ICU: treatment in the intensive care unit, ARDS: acute respiratory distress syndrome, Oxygen: required oxygen therapy in a number of subjects. *Significant at 5% level

Presence of known factors		Antibody response, n (%)		p value *
		Positive	Negative	
Hospitalized (n= 241)	Yes	107 (78.7)	29 (21.3)	0.145
	No	90 (85.7)	15 (14.3)	
ARDS (n = 196)	Yes	25 (96.2	1 (3.8)	0.137
	No	144 (84.7)	26 (15.3)	
ICU (n = 68)	Yes	19 (95.0)	1 (5.0)	0.664
	No	42 (87.5)	6 (12.5)	
Oxygen (n = 160)	Yes	33 (97.1)	1 (2.9)	0.022^*^
	No	102 (81.0)	24 (19.0)	

**Table 4:** Relation between antibody response and time after the onset of infection in weeks n=number of subjects. *Significant at 1% level

Time after the onset of infection (weeks)	Antibody response, n (%)		p value*
	Positive	Negative	
2-4	51 (89.5)	6 (10.5)	Reference
4-8	68 (85.0)	12 (15.0)	0.445
8-12	43 (69.4)	19 (30.6)	0.007^*^
12-17	35 (83.3)	7 (16.7)	0.371

## Discussion

The first confirmed case of COVID-19 in Kerala was reported on January 30, 2020, marking the commencement of the pandemic in the state. Despite effective initial containment, a surge in cases by March 2020 indicated the start of the first wave, initially driven by residents returning from abroad and other Indian states. The pandemic’s progression in Kerala was characterized by several waves, each with distinct peaks in case numbers [6].

From October 2020 to September 2021, Kerala faced multiple COVID-19 waves and the emergence of various virus variants, including Alpha (B.1.1.7), Beta (B.1.351), Gamma (P.1), and Delta (B.1.617.2), known for their increased transmissibility and potential impact on vaccine efficacy [7]. The state’s vaccination campaign began on January 16, 2021, initially targeting healthcare and frontline workers, then expanding to older adults and those with comorbidities, and eventually the wider population [8]. By October 20, 2021, over 30 million vaccine doses had been administered, with 94% of the eligible population having received at least one dose and 47% fully vaccinated. Kerala’s seroprevalence was reported at 44.4% in May 2021 by the Indian Council of Medical Research (ICMR) in the community, which was lower than the national average of 67.6%, reflecting the serological profile of the state during the pandemic [9,10].

In this study, we characterized the IgG response to the S1 subunit of the SARS-CoV-2 spike protein in patients from Kerala, India, during the initial wave of the COVID-19 pandemic and before the free availability of vaccines (from October 2020 to September 2021). The research was conducted at Government Medical College, Kozhikode, a tertiary care center, involved measuring IgG levels in serum samples from 258 patients at various time points following symptom onset. The analysis focused on understanding the dynamics of the IgG response over time and its association with comorbidities. Subsequently, the correlation between IgG responses and clinical parameters was examined providing insights into the immune response elicited by the virus in a diverse patient population.

The results indicate an increased positivity rate of IgG antibody response (76.4%) among the study participants, suggesting a robust immune reaction to SARS-CoV-2 infection in this population to the initial infection from a new viral outbreak. The antibody response varied over the severity of illness, with higher rates observed in patients with more severe manifestations of COVID-19. This observation aligns with previous studies indicating a correlation between disease severity and antibody response [11,12].

The S1 domain of the spike protein contains the receptor-binding domain (RBD) that mediates the interaction of the virus to the host cell receptor, angiotensin-converting enzyme 2 (ACE2) [13]. The RBD is a major target of neutralizing antibodies that can block the virus’ attachment to the host cell and prevent infection, resulting in a better clinical outcome [14,15]. Using an ELISA kit that recognizes IgG antibodies to the entire S1 domain, encompassing the RBD and other epitopes, we measured the IgG response in patients with different disease severity. We found that patients with severe disease had higher IgG levels than those with mild or asymptomatic disease, consistent with prior reports [16]. This suggests that the IgG response reflects the viral load and the inflammatory response associated with COVID-19 infection in patients.

We also found that the IgG response was varying after the first infection period. The IgG response peaked at two to four weeks and four to eight weeks after the onset of infection. However, the IgG response showed an increasing trend again at >= 12 weeks, which may indicate a secondary exposure or a memory response in the population, associated with another wave of COVID-19, such as Delta [17]. Previous studies have shown that the IgG response against SARS-CoV-2 can persist for several months after infection but may vary depending on the assay and the antigen used [18]. We measured IgG antibodies to the S1 domain of the virus using an ELISA kit, which might not reflect the complete antibody response to the virus [19]. Assessing IgG response to other viral antigens could provide additional insights. In a parallel study conducted concurrently, genomic surveillance revealed significant alterations in the virus over the final seven months of the study period. Notably, variants such as Delta were observed to be the predominant strain during this period [20,21]. This may also induce an increasing trend of IgG at >= 12 weeks.

We also examined the effect of comorbidities on the IgG response. We found that the IgG response was not significantly different between patients with or without diabetes mellitus (DM), hypertension (HTN), or coronary artery disease (CAD). The association between the above comorbidities and IgG response is unclear, as some studies report lower IgG levels in patients with DM or HTN, while others find no correlation [22-24].

Finally, we analyzed the clinical outcomes of the IgG response among patients. We found that the IgG response was higher in patients who required hospitalization, oxygen therapy, ICU admission, or developed acute respiratory distress syndrome (ARDS) than in those who did not. This is in line with previous studies that showed that the IgG response was associated with the disease severity and the need for intensive care [25]. It is possible that the IgG response is a marker of the immune activation, rather than a causal factor of the disease outcome. Depending on the viral load, host factors, IgG response, and disease severity may vary. Early and high IgG response may confer protection by neutralizing the virus and preventing infection, while late high or low IgG response may enhance the infection by causing inflammation or severity of infection [26]. Therefore, to determine the role of IgG in COVID-19 disease progression or suppression requires additional studies. It should also be mentioned that this study was done on data collected from direct interviews with recovered patients, as well as hospital discharge summaries and follow-up records, which included information on ICU stay and oxygen use.

This study substantiates the humoral immunity and possible protection against SARS-CoV-2 infection, but further studies are required to confirm our results and investigate the IgG response to other viral antigens and assays, as well as its function and mechanism in COVID-19.

This study has some drawbacks that should be acknowledged. The sample size in the study is small and may not reflect all COVID-19 patients during the period of study. The study was cross-sectional, and patients were not tracked over a period of time to test antibody response for a longer time. The study used only one ELISA kit that specifically binds to a region of the SARS-CoV-2 virus, which may not be the same by other methods of assay. The study did not examine other immune responses that could prevent reinfection or measure other factors such as viral load, variants, vaccination, previous coronavirus exposure, etc., which may affect the antibody response in the subjects studied.

This type of study may facilitate the development of enhanced immunization protocols and contribute to the optimization of vaccine strategies through detailed analysis of immune responses [27]. Hence, the present study may offer a foundation for future investigations into vaccine-induced immunity. The observations derived from this study may be of use to understand the immune response to a new unknown infection of the pandemic potential, and the disease spectrum could behave differently in patients infected when a similar pandemic strikes in the future.

## Conclusions

The study conducted in Kerala has demonstrated a robust IgG seropositivity rate of 76.4% in response to SARS-CoV-2, indicating a strong humoral immune response. Notably, IgG titres were observed to correlate with the severity of the disease, exhibiting peak levels at intervals of two to four weeks, four to eight weeks, and ≥12 weeks post-infection. This temporal pattern suggests that IgG titres could serve as potential biomarkers for immune activation and prognostication of the disease trajectory. Interestingly, the presence of comorbid conditions did not significantly alter IgG levels, underscoring the uniformity of the immune response across diverse patient profiles. These initial findings provide a compelling basis for further research to elucidate the implications of IgG dynamics in COVID-19 disease management and outcome prediction.
